# Supplementary material for: Ultrahigh thermoelectric power factor in flexible hybrid inorganic-organic superlattice
Source: Nat Commun. 2017 Oct 18;8:1024. doi: 10.1038/s41467-017-01149-4 (PMC5647338; doi:10.1038/s41467-017-01149-4)
Supplement: Supplementary file 1 — Supplementary Information [file 41467_2017_1149_MOESM1_ESM.pdf]

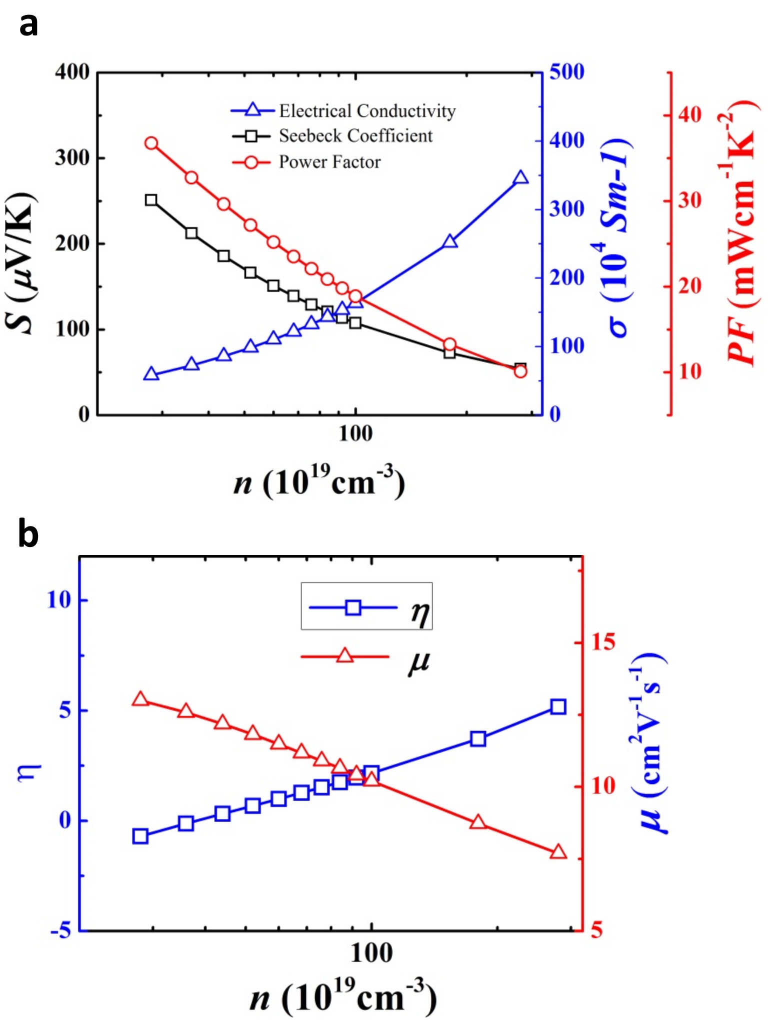

Supplementary Figure 1. (a) The estimated dependence of Seebeck coefficient (*S*), electrical conductivity (σ) and power factor *(PF)* on the carrier concentration of organic-intercalated TiS_2. **(b)** The dependence of the reduced Fermi Level (η) and the mobility on the carrier concentration of the organic-intercalated TiS_2. The details of the estimation are shown in the supplementary discussion on page 12-13.

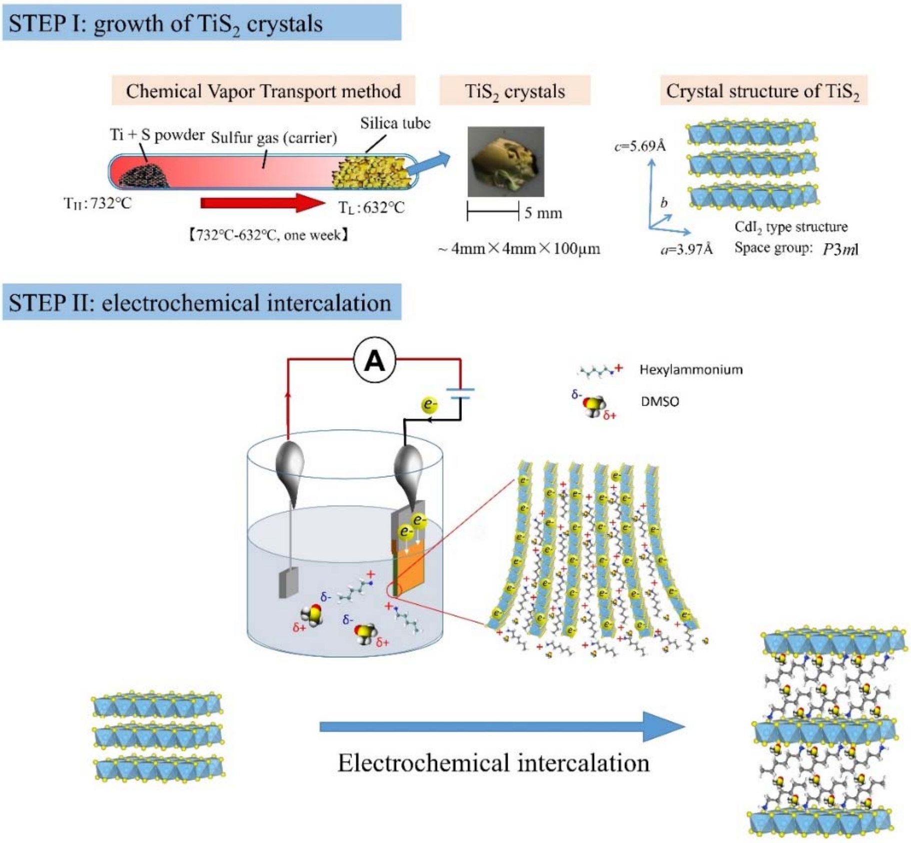

Supplementary Figure 2. Schematic of the fabrication process of the TiS_2 crystal and TiS_2/organic superlattice by chemical vapor transport (CVT) method and electrochemcial intercalation method. TiS_2 has a *CdI_2* structure with space group P-3m1. It is a quasi-2-dimensional structure, where the layers are connected by the van der Waals gap. The TiS_2 single crystal was obtained by a standard CVT method. Titanium and sulfur powder mixture was sealed in evacuated silica tube. The silica tube was horizontally placed with the powders put at one end. Then the tube was put into a three-zone furnace, in which a temperature gradient across the tube was established. The optimized temperature for the hot end and cold end are 732 ºC and 632 ºC, respectively. Additional sulfur was added as the agent which carries the vapor of Ti and Sulfur form the hot end to the cold end, where the single crystal of TiS_2 was obtained.

To fabricate the TiS_2/organic layered material, an electrochemical intercalation process was used, which is very similar to the electrochemical reaction of the lithium

ion battery electrodes. When the voltage is applied, the TiS_2 layers were negatively charged and the positive organic cations were intercalated into the van der Waals gaps driven by the electrostatic force, forming a layer by layer structure. The typical voltage is -1.5 V, and the electrolyte concentration is 0.5 M and the electrochemical reaction time is 15 minutes.

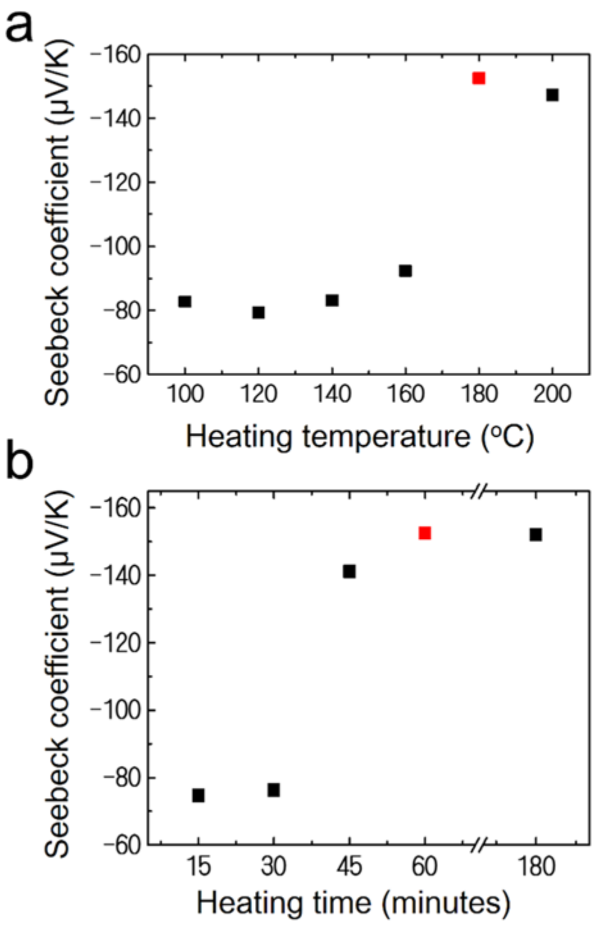

Supplementary Figure 3. (a) The dependence of the Seebeck coefficient of $TiS_2[HA]_x[DMSO]_y$ on the temperature of vacuum heating for 1-hour heating. The Seebeck coefficient is closely related to the carrier concentration. The sudden increase of Seebeck coefficient at 180°C suggests a drastic decrease of carrier concentration. **(b)** The dependence of the Seebeck coefficient of $TiS_2[HA]_x[DMSO]_y$ on time for the vacuum heating at 180°C.

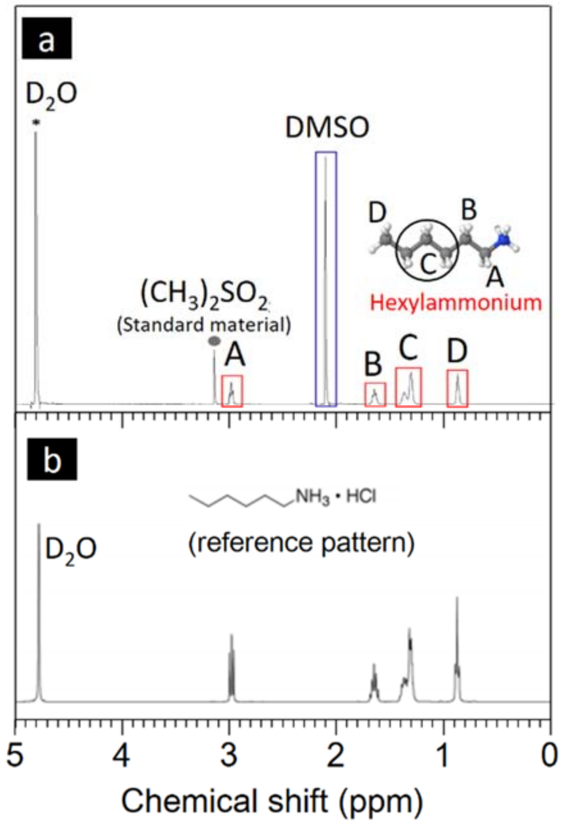

Supplementary Figure 4. ^{1}H NMR spectrum of (a) $TiS_2[HA_x]$ and (b) $NH_3{\cdot}HCl$ dissolved in D_2O. (reference 1)

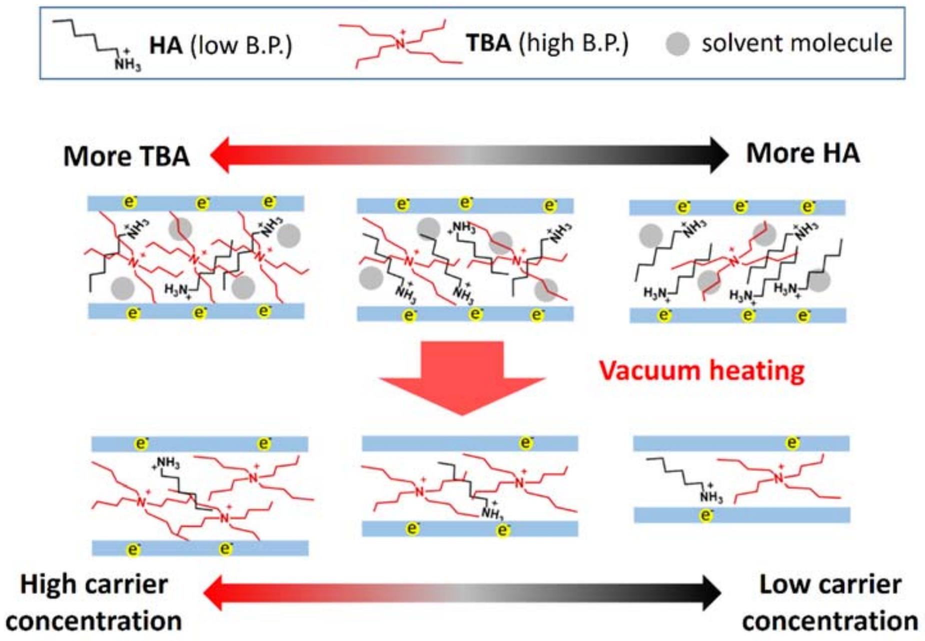

Supplementary Figure 5. Illustration on carrier concentration tuning mechanism in the hybrid inorganic/organic superlattice. Hexylammonium ion (HA) is selected as the lower boiling point (B.P.) molecule and tetrabutylammonium (TBA) is selected as the higher boiling point molecules. Firstly, TBA and HA with different ratios are co-intercalated into the interlayer spacing of single crystalline TiS_2 together with the neutral solvent molecules. Equal amounts of electrons in the TiS_2 layers are generated to compensate the positive charges of the TBA and HA cations due to the electrical neutrality. Then the hybrid inorganic/organic superlattices are vacuum heated at an intermediate temperature between the boiling points of HA and TBA. HA with a lower boiling point is almost completely evaporated and the TBA with a high boiling point can be maintained to sustain the hybrid superlattice structure. By changing the ratio between TBA and HA, the remaining density of cations can be adjusted and therefore the corresponding electron concentration in the TiS_2 layers can be tuned. For all of the ratios, the immobile TBA cations are the key to maintain the stage-1 inorganic-organic superlattice structure.

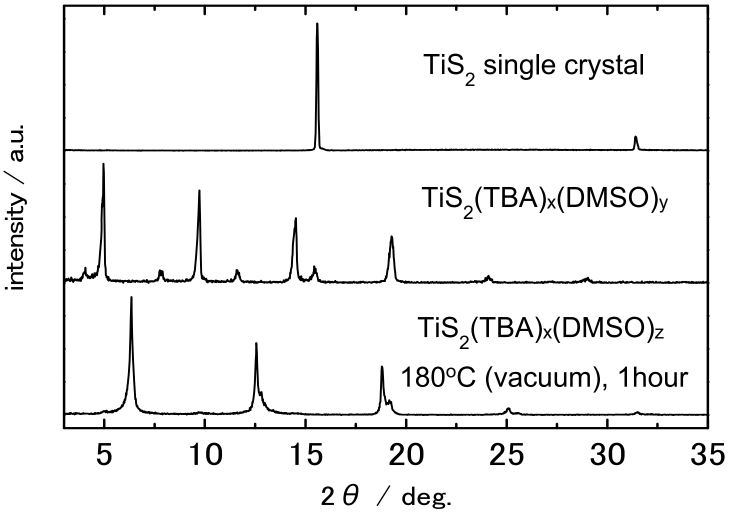

Supplementary Figure 6. XRD patterns of single crystalline TiS_2, TiS_2 intercalated with TBA, and then vacuum heated at 180 °C for 1 hour. The shift of peaks is due to the evaporation of the solvents DMSO, which results in a percentage loss from y to z and a shrinkage of interlayer distance. The Seebeck coefficient slightly changed from -59.6 μV/K to -63.8 μV/K after vacuum heating, suggesting slight decrease of carrier concentration due to the corresponding evaporation of TBA cations.

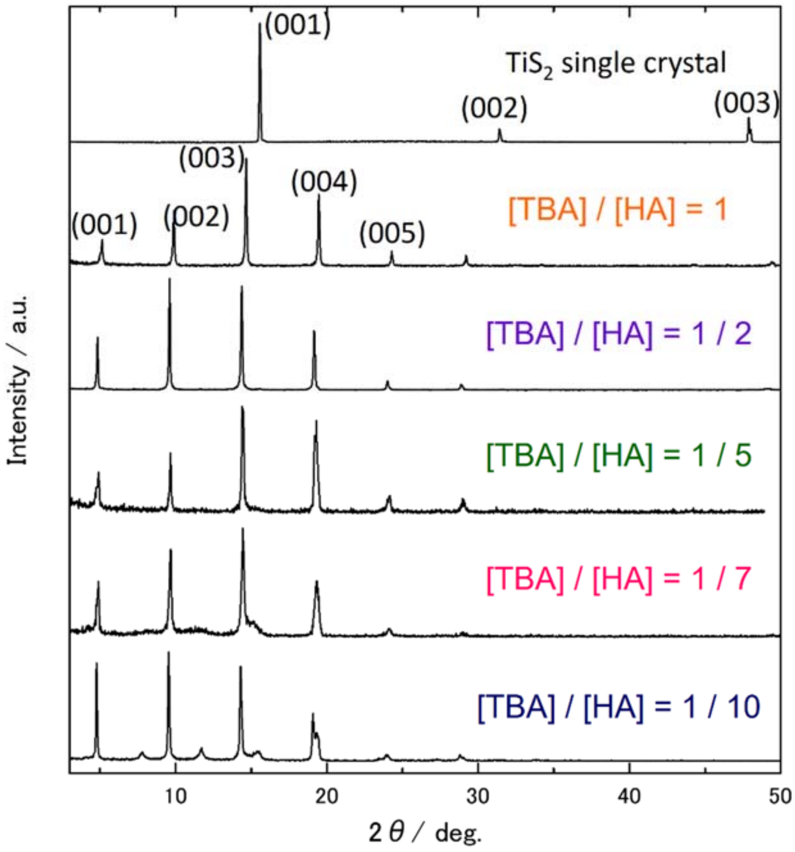

Supplementary Figure 7. XRD patterns of single crystalline TiS_2 and the hybrid $TiS_2(TBA)_x(HA)_y(DMSO)_z$ fabricated by electrochemical intercalation with different nominal [TBA]/[HA] ratios in the electrolyte solution.

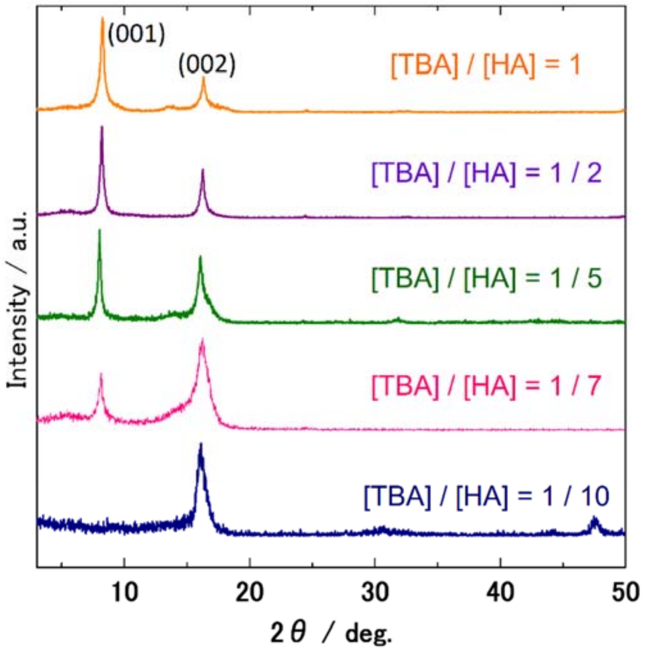

Supplementary Figure 8. XRD patterns of the electrochemically synthesized $TiS_2(TBA)_x(HA)_y(DMSO)_z$ samples with different nominal [TBA]/[HA] ratios in the electrolyte solution after vacuum heating at 180°C for 1 hour.

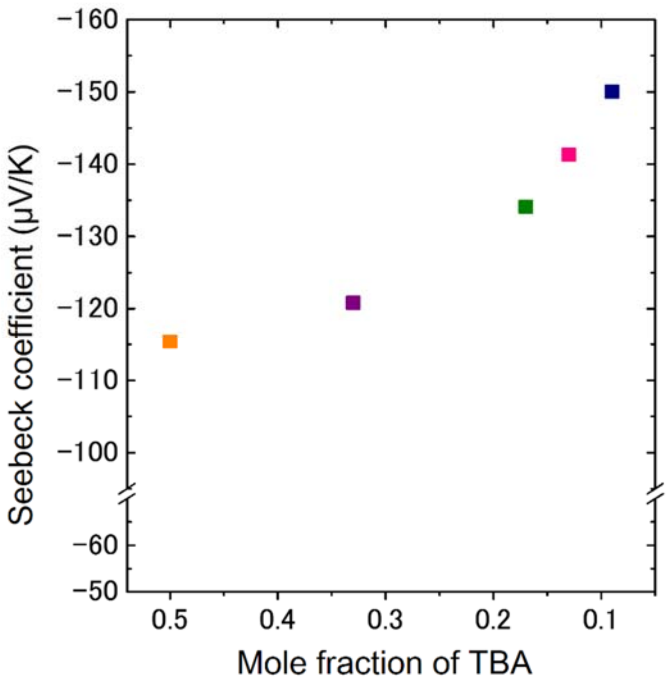

Supplementary Figure 9. The Seebeck coefficients of the vacuum heated $TiS_2(TBA)_x(HA)_y(DMSO)_z$ samples with different nominal [TBA] mole fractions ([TBA]/[TBA]+[HA]). Theoretically, the [TBA]/[HA] ratio can be changed from positive infinity to negative infinity with great tunability of carrier concentration. However, in this paper, the ratios from 1:1 to 1:7 were tried with the obtained Seebeck coefficient varying from -115 to -142 μV/K (The 1:10 composition is excluded here, as the content of TBA is too few to support the stage-1 structure after evaporation of the HA molecules, which is confirmed in Fig. S7). If large [TBA]/[HA] ratios, from 10:1 to 1:1, were tried, only a small amount of HA molecules will be removed and the carrier concentration will still remain very high. The Seebeck coefficient will be lower than those with smaller [TBA]/[HA] ratios. The extreme case is $TiS_2[TBA]_x$, which shows a Seebeck coefficient of -63.8 μV/K. In other words, by varying the [TBA]/[HA] ratio, the Seebeck coefficient can be tuned from -63.8 μV/K to -142 μV/K, which is indeed a very wide range for the optimization of thermoelectric properties.

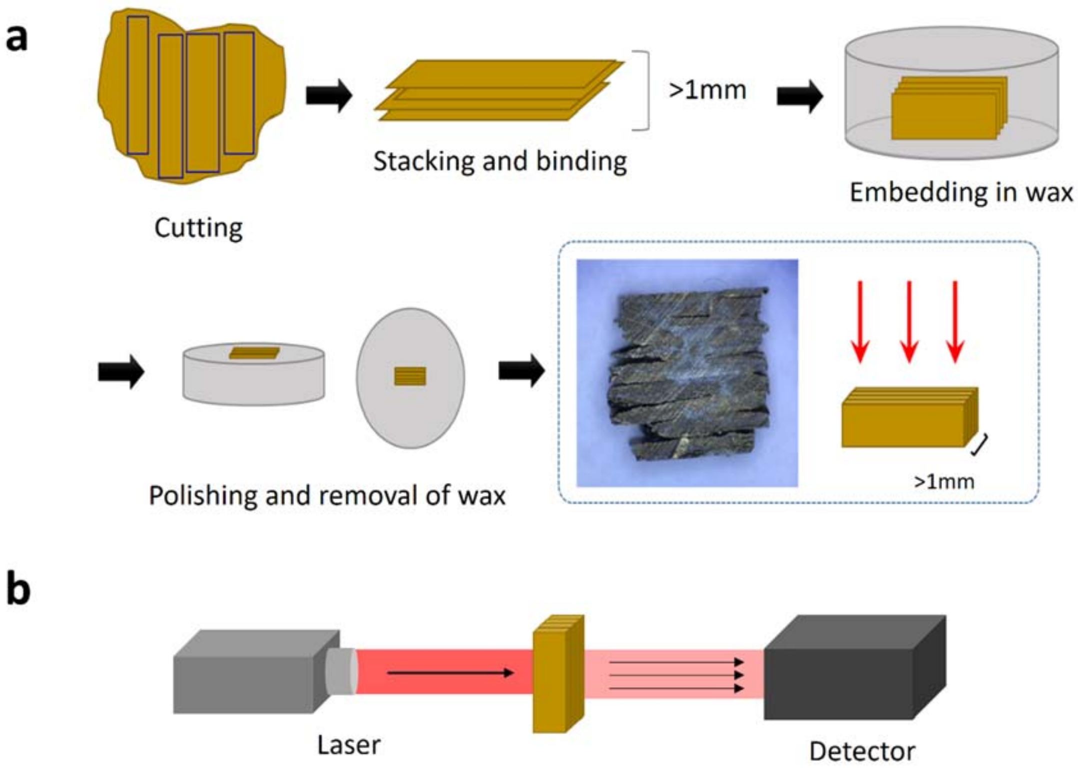

Supplementary Figure 10. Sample preparation for the in-plane thermal diffusivity measurement by the laser flash method. (a) The thin sample (4mm×4mm×150μm) was first cut into many small pieces and then stacked and bonded using epoxy resin. It was then put into melted wax and solidified. Both sides of the sample were polished using a sand paper. The wax was removed by heating and then washing in ethyl ether. (b) Thermal diffusivity of the prepared sample was measured by a standard laser flash analysis equipment (ULVAC, TC-9000).

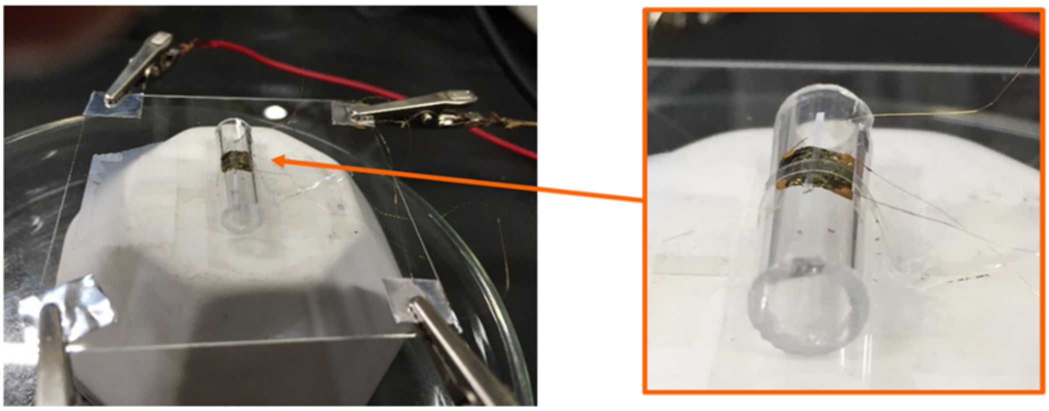

Supplementary Figure 11. The sample (4mm×4mm×150μm) was firmly attached onto the surface of glass tubes with different radii. The electrical resistance was measured using the van der Pauw method as a function of the bending radii, namely, the radii of the glass tube.

Supplementary Note 1. Dependence of the thermoelectric properties on the carrier concentration of the TiS_2–based inorganic-organic superlattice

For pure n-type TiS_2 single crystals, the carrier concentration is ~ 2.8*10^{20} cm^{-3}.[2] During the electrochemical process, the TiS_2 layers would be electrochemically reduced and the negative electron charge was balanced by the intercalated organic cations (e.g. hexylammonium) according to the requirement for electrical neutrality. The effective carrier concentration in the hybrid materials would increase after electrochemical intercalation. Given an increase of carrier concentration from 2.8*10^{20} cm^{-3} to 2.8*10^{21} cm^{-3}, the corresponding Seebeck coefficient can be estimated according to the following equation,

$$S = \frac{8\pi^2 {k_B}^2}{3eh^2} m^* T \left(\frac{\pi}{3n}\right)^{\frac{2}{3}} \quad (1),$$

where, n is the carrier concentration and m* is the effective mass of the carrier. Assuming that the intercalation does not affect the effect mass of the carrier (estimated to be ~ 4.8 m_e [3]), the Seebeck coefficient (absolute value) was reduced monotonously from 251 μV/K to 54 μV/K, after intercalation, as shown in Fig. S1(a).

The positive ions (e.g. hexylammonium) could potentially scatter the electrons inside the TiS_2 layers due to the Coulomb interaction. The drift mobility μ for acoustic phonon scattering and arbitrary electron degeneracy can be expressed by equation (2) according to ref.3, where v_L is the velocity of longitudinal sound waves, ρ is the sample density, Ξ is deformation potential, m^* is effective mass of carriers, the above parameters are assumed to be unchanged after intercalation herein. The drift mobility μ for acoustic phonon scattering and arbitrary electron degeneracy can be expressed by equation (4) in ref.4, where the change of sample density is neglected. The μ only depends on the reduced Fermi level η, which can be estimated by equation 3 and 4. The carrier concentration dependent η and μ is shown in Fig. S1(b).

$$\mu^{ph} = \frac{\sqrt{2} e\pi h^4}{3(k_B T)^{3/2}} \frac{v_L^2 \rho}{\Xi^2 (m^*)^{5/2}} \frac{F_0(\eta)}{F_{1/2}(\eta)} \quad (2)$$

$$S = -\frac{k_B}{e}\left(\frac{(2+\lambda)F_{1+\lambda}(\eta)}{(1+\lambda)F_\lambda(\eta)} - \eta\right) \quad (3)$$

$$F_j(\eta) = \int_0^{\infty} \frac{\xi^j d\xi}{1+\exp(\xi-\eta)} \quad (4)$$

The electrical conductivity of the hybrid superlattices can be then calculated using $\sigma = n\mu q$. Due to the increase of the carrier concentration, the electrical conductivity was increased monotonously from $58*10^4$ Sm^{-1} to $345*10^4$ Sm^{-1}, as shown in Fig. S1. The power factor thus decreases from 37 to 10 mW cm^{-1} K^{-2}. However, it remains high when the carrier concentration does not change too much.

Supplementary Reference

1. Wittenberg J.B., Zavalij P.Y., Isaacs L., Supramolecular Ladders from Dimeric Cucurbit[6]uril. *Angew. Chem. Int. Edit.*, **52**, 3690-3694(2013)

2. Imai H., Shimakawa Y., & Kubo Y., Large thermoelectric power factor in TiS_2 crystal with nearly stoichiometric composition, *Phys. Rev. B*, **64**, 241104(2004).

3. Amara A., Frongillo Y., Aubin M. J., & Jandl S., Thermoelectric power of TiS_2, *Phys. Rev. B*, 36, 6416.

4. Fu C., Zhu T., Pei Y., Xie H., Wang H., Snyder G. J., Liu Y., Liu Y., & Zhao X., High Band Degeneracy Contributes to High Thermoelectric Performance in *p*-Type Half-Heusler Compounds, *Adv. Energy Mater.* **4**, 1400600.(2014)
